# Endogenous Ovarian Angiogenesis in Polycystic Ovary Syndrome-Like Rats Induced by Low-Frequency Electro-Acupuncture: The CLARITY Three-Dimensional Approach

**DOI:** 10.3390/ijms19113500

**Published:** 2018-11-07

**Authors:** Tong Ma, Peng Cui, Xiaoyu Tong, Wei Hu, Linus R. Shao, Feifei Zhang, Xin Li, Yi Feng

**Affiliations:** 1 Department of Integrative Medicine and Neurobiology, School of Basic Medical Sciences, Institutes of Brain Science, Brain Science Collaborative Innovation Center, State Key Laboratory of Medical Neurobiology, Institute of Acupuncture and Moxibustion, Fudan Institutes of Integrative Medicine, Fudan University, Shanghai 200032, China; 15211010074@fudan.edu.cn (T.M.); 13211010081@fudan.edu.cn (P.C.); 17211010071@fudan.edu.cn (X.T.); 15301050259@fudan.edu.cn (W.H.); 2 Shanghai Key Laboratory of Acupuncture Mechanism and Acupoint Function, Shanghai 200032, China; 3 Department of Physiology/Endocrinology, Institute of Neuroscience and Physiology, The Sahlgrenska Academy, University of Gothenburg, 40530 Gothenburg, Sweden; Ruijin.Shao@fysiologi.gu.se; 4 Department of Obstetrics and Gynecology, Shanghai Key Laboratory of Female Reproductive Endocrine Related Diseases, Shanghai Medical School, Fudan University, Shanghai 200011, China; feifeizhang@fudan.edu.cn

**Keywords:** PCOS-like model, electro-acupuncture, ovarian vascularity and neo-angiogenesis, CLARITY

## Abstract

We sought to determine the role of ovarian vascularity and neo-angiogenesis in the development of mature follicles in polycystic ovary syndrome (PCOS) and to identify any changes induced by low-frequency electro-acupuncture (EA). Twenty-eight 21-day-old female Wistar rats were randomly divided into four groups—Control, Obesity, PCOS-like, and PCOS-like-EA (n = 7/group). Rats in the Obesity group were fed a high-fat diet throughout the experiment. Rats in the PCOS-like and PCOS-like-EA groups were implanted with a sustained-release tube containing 5α-dihydrotestosterone (DHT) beneath the skin of the neck. Rats in the PCOS-like-EA group received low-frequency EA treatment starting at 70 days for 30 min five times a week for four weeks. At the end of the experiment, all rats were euthanized and perfused with hydrogel. The ovaries were collected for clarification and imaging, and ovarian vascularity and neo-angiogenesis were analyzed. Compared with Control and Obesity rats, the ovaries in DHT-induced PCOS-like rats were smaller in size and had fewer mature follicles and corpora lutea. EA increased angiogenesis in the antral follicles of PCOS-like rats, which in turn promoted follicle maturation, ovulation, and CL formation. Therefore, endogenous ovarian angiogenesis plays a very important role in follicular maturation and might be one of the peripheral and direct mechanisms of EA on PCOS.

## 1. Introduction

Polycystic ovary syndrome (PCOS) is a complex endocrine and metabolic syndrome in women of reproductive age with an incidence rate of about 3–26% [1,2,3]. The 2004 Rotterdam Criteria suggest that PCOS should be diagnosed by at least two of the following three criteria: oligo/anovulation, clinical or biochemical hyperandrogenism, and polycystic ovaries by ultrasound. In 2012, the Androgen Excess and PCOS Society criteria recommended that PCOS should be defined as clinical or biochemical hyperandrogenism associated with ovulatory dysfunction in the form of oligo-anovulation or polycystic ovaries [4]. According to the above two sets of criteria, disorders of follicle maturation and anovulation are the most typical symptoms of PCOS. Even with the help of in vitro fertilization, including embryo transfer technology with high doses of exogenous hormones, the majority of PCOS patients are still unable to produce mature follicles [5].

Both pre-clinical and clinical studies suggest that follicular development and ovulatory disorders are associated with ovarian vascularity [6]. For example, the construction of follicle-vasculature relationship maps shows age- and gonadotropin-dependent increases in vascularity and branching surrounding ovarian follicles [6] and unilateral intrabursal injection of axitinib, a vascular endothelial growth factor (VEGF) receptor-targeted tyrosine kinase inhibitor, retards the neo-angiogenesis that is associated with defective ovulation [6]. Abnormal expression of multiple angiogenic factors is seen in ovarian tissues collected from PCOS patients, including VEGF, angiopoietin-1 and -2, platelet derived growth factor (PDGF), transforming growth factor beta (TGF-β), and basic fibroblast growth factor (bFGF). These angiogenic factors are likely involved in the pathophysiology of PCOS [7]. In PCOS patients, hyper-vascularized and excessive blood vessels are found in the membrane cell layer and the interstitium of ovarian cystic follicles [8,9] and the expression of VEGF is significantly increased in the follicular stroma [10]. Furthermore, dehydroepiandrosterone (DHEA)-induced PCOS-like rats exhibit increased circulating and ovarian VEGF levels, and treatment with VEGF receptor inhibitor reduces the numbers of ovarian cystic follicles in these PCOS-like rats [11]. However, that animal study failed to show whether the elevated ovarian VEGF level was derived from interstitial cells or from follicles. Also, although the VEGF receptor inhibitors can reduce the numbers of cystic follicles, they do not lead to further development into mature follicles.

As a traditional natural therapy, acupuncture has a long history of use for the treatment of gynaecological disorders in China. It has been confirmed that acupuncture can regulate the central nervous system through the hypothalamus-pituitary-ovary axis [12], and it also directly affects the peripheral tissues such as the ovary, adrenal gland, and adipose tissue [13,14]. In the clinic, acupuncture has been proven to have a positive effect on the development of follicles and the promotion of ovulation in infertile women [15], and low-frequency electro-acupuncture (EA) with local and distal acupoints promotes ovulation and increases the pregnancy success rate [16]. Our previous studies showed that the rate of ovulation was as high as 38% after 14 sessions of acupuncture treatment in PCOS patients, which was similar to that of clomiphene, a first-line anti-PCOS drug. The pregnancy rate was improved [17], although there was no significant increase in the live birth rate [18].

In the present study, female Wistar rats were used to set up the PCOS-like model induced by 5α-dihydrotestosterone (DHT). The PCOS-like-EA group was treated with low-frequency EA bilaterally at the “Sanyinjiao” (SP6) and “Guilai” (ST29) acupoints. Ovaries were collected for clarification and subsequent three-dimensional quantitative analysis. The numbers of different types of follicles and their relationship with the local vasculature were analyzed to study the effect of EA on angiogenesis in PCOS.

## 2. Results

### 2.1. Pathological Manifestations in DHT-Induced PCOS-Like Rats and the Effect of EA

**Figure 1:**
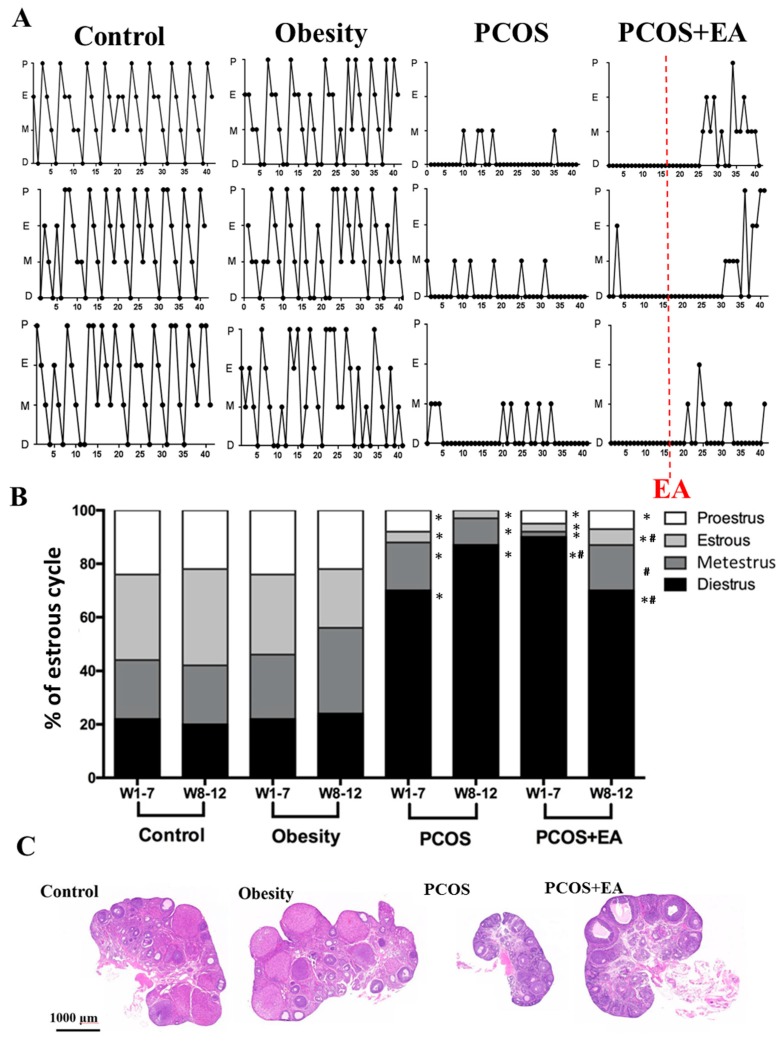
Pathological manifestations of the 5α-dihydrotestosterone (DHT)-induced polycystic ovary syndrome (PCOS) rat model and the effect of electro-acupuncture (EA). (**A**) Three typical oestrous cycle charts of rats from each group, P: Proestrus; E: Oestrus; M: Metestrus; D: Diestrus. (**B**) The ratio of Proestrus, Oestrus, Metestrus, and Diestrus stage to all observed oestrous cycles. All values are the mean ± standard error; * *p* < 0.05 vs. control group; ^#^ *p* < 0.05 vs. PCOS group. (**C**) Hematoxylin and eosin staining of paraffin-embedded sections from each group.

After implantation of DHT, the PCOS-like and PCOS-like-EA rats developed obvious PCOS-like features. As shown in Figure 1A, three representative graphs of oestrous cycles in each group are shown. Although compared with the Control group, the proportions of rats with diestrus phase in the PCOS-like and PCOS-like-EA groups were significantly increased, 85% of the rats in the PCOS-like-EA group had restored oestrous cycles. We analyzed the oestrous cycle (Figure 1B) and the proportions in each phase of the oestrous cycle in the Control and Obesity groups showed that these rats had regular 4- or 5-day cycles. However, the PCOS-like rats remained mostly at the diestrus or metestrus stages. Compared with the PCOS-like group, the proportion of rats in the diestrus stage in the PCOS-like-EA group was significantly decreased from 90 to 70%. In addition, hematoxylin and eosin staining showed that the ovaries in PCOS-like rats were smaller compared to the Control and Obesity groups, with fewer mature follicles and CL. Compared with the PCOS-like group, ovaries of the PCOS-like-EA group were larger, with more preovulatory follicles and CL (Figure 1C). Taken together, these results indicated that EA could partially restore ovarian morphology and function in PCOS-like rats.

### 2.2. Quantitative Analysis of Follicles and CL in Ovaries Using the CLARITY Methods.

**Figure 2:**
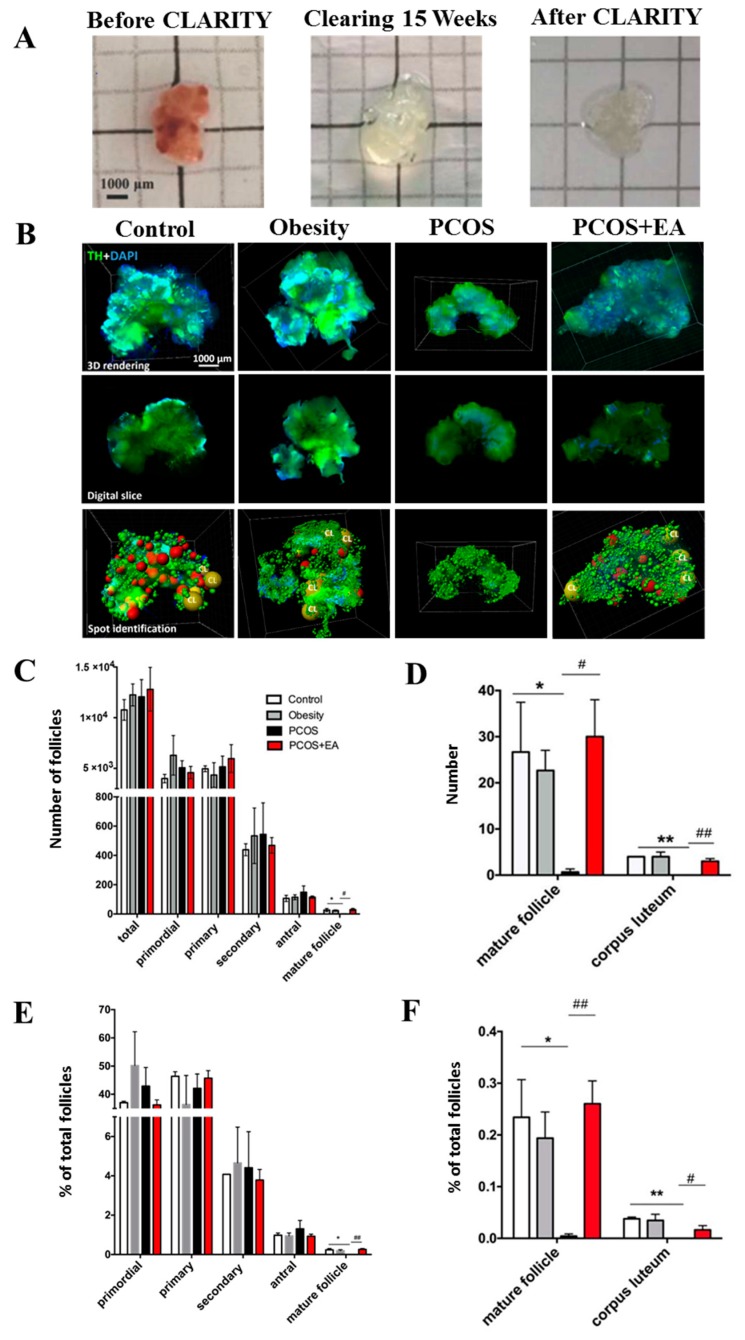
CLARITY shows that EA increases the numbers of mature follicles and Cl in the ovaries from PCOS-like rats. (**A**) The clarification process of a rat ovary. (**B**) Ovaries from rats in the different groups after CLARITY processing and immunostaining using specific antibodies followed by data transformation using the Imaris Spot algorithm. Upper panels: 3D rendering of whole ovary images; middle panels: digital slices showing follicles at individual planes; lower panels: Data transformation into spot graphs following the identification of follicles using specific markers. Background staining using tyrosine hydroxylase antibodies provided outlines for the identification of all follicles. Red spots represent preovulatory follicles. CL, corpus luteum. (**C**) The numbers of follicles at different developmental stages in the different groups. (**D**) The numbers of mature follicles and corpora lutea. (**E**) The ratio of the different follicle stages in each group. (**F**) The ratio of mature follicles and CL. * *p* < 0.05 and ** *p* < 0.01 vs. the Control group; ^#^ *p* < 0.05 and ^##^ *p* < 0.01 *vs*. the PCOS group.

Ovaries began to become transparent after 8–12 weeks passive clearing (Figure 2A), and this allowed us to calculate the numbers of different kinds of follicles and their proportions (Figure 2B). As shown in Figure 2C, there were about 25 preovulatory follicles per ovary in the Control group and about 20 preovulatory follicles per ovary in the Obesity group, but none in the PCOS group (*p* < 0.05 compared with the Control group). After 4 weeks of EA treatments (Figure 2C,D), there was an average of 25 follicles per ovary in the PCOS-like-EA group (*p* < 0.05 compared with the PCOS-like group). There were about three CL in the Control group and Obesity group, 0 in the PCOS-like group (*p* < 0.01 compared with the Control group), and 2 in the PCOS-like-EA group (*p* < 0.01 compared with the PCOS-like group).

The ratios of different kinds of follicles to total follicles were also analyzed (Figure 2E,F). The proportions of preovulatory follicles in the Control and Obesity groups were on average 0.25% of the total follicles, while the proportion in the PCOS-like group was 0%. After EA treatment, the proportion of preovulatory follicles in the PCOS-like-EA group reached 0.25%, which was the same as the Control group and was significantly higher than that of the PCOS-like group (*p* < 0.05). Only about 0.03% of the total follicle population consisted of CL in the Control and Obesity groups, and no CL were seen in the PCOS-like group. The proportion of CL increased to 0.02% in the PCOS-like-EA group, which was obviously higher than the PCOS-like group (*p* < 0.01). Thus we conclude that EA can promote follicular maturation and ovulation in DHT-induced PCOS-like rats.

### 2.3. EA Promotes Angiogenesis in PCOS-Like Ovaries

**Figure 3:**
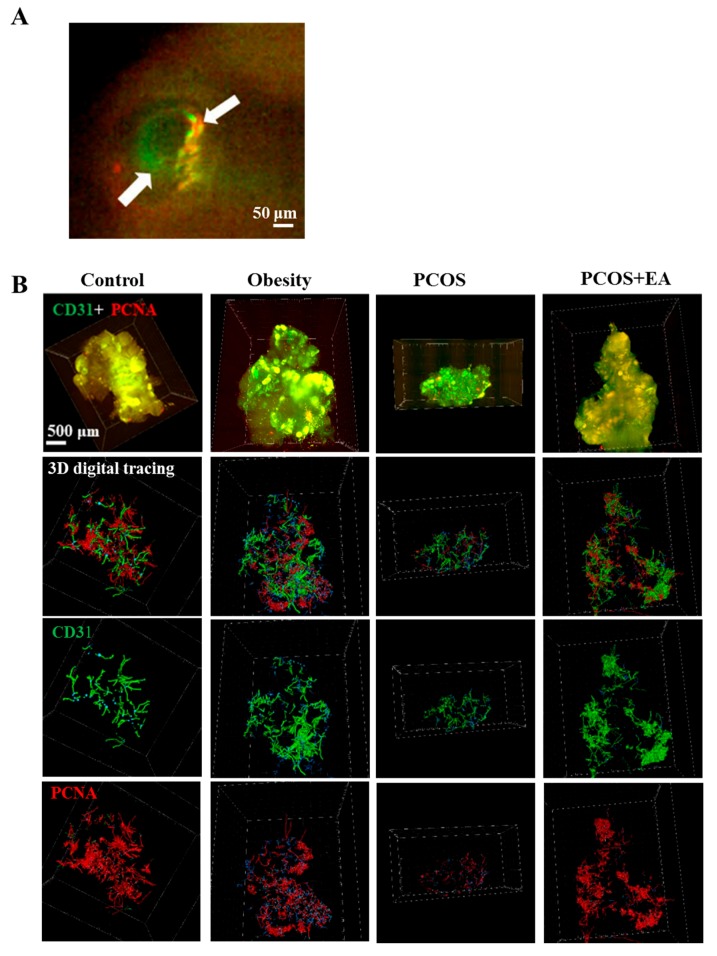
EA promotes angiogenesis in PCOS-like rat ovaries. (**A**) CD31 was used to stain the vasculature, and proliferating cell nuclear antigen (PCNA) was used to stain the neovasculature. The white arrows indicated the co-localization of PCNA with CD31 in one follicle. (**B**) Ovaries from rats from different groups after CLARITY processing and immunostaining using specific antibodies, followed by data transformation using the Imaris Filament algorithm. First row: 3D rendering of whole ovary images; second row: vasculature and neovasculature within the ovaries; third row: vasculature within each ovary; fourth row: neovasculature in each ovary.

**Figure 4:**
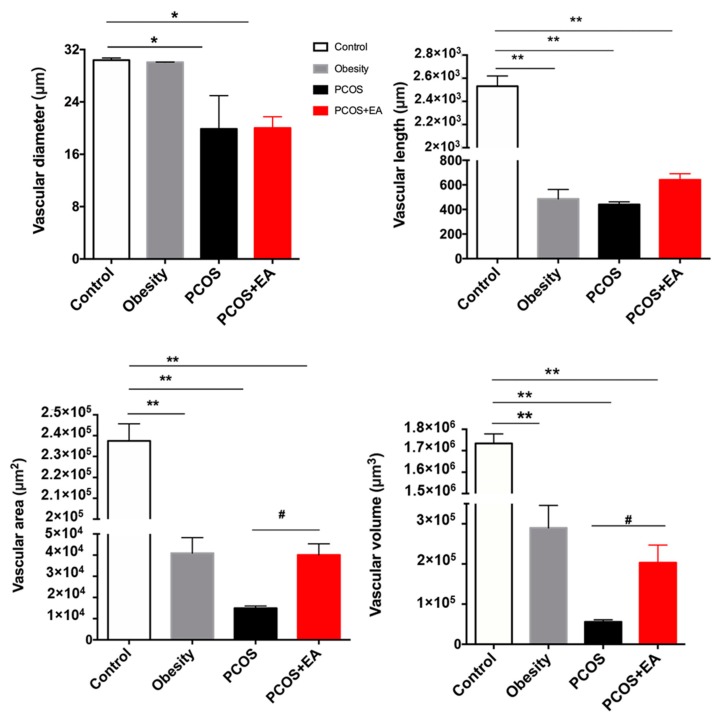
Quantification of ovarian vasculature using the Imaris software. Quantification of the diameter, length, area, and volume within the ovary from each group. * *p* < 0.05 and ** *p* < 0.01 vs. the Control group; ^#^ *p* < 0.05 vs. the PCOS group.

Platelet endothelial cell adhesion molecule 1 (PECAM-1, CD31) was used to show ovarian vascularity, and proliferating cell nuclear antigen (PCNA) was used to specifically mark neovascularity (Figure 3A). The Imaris filament algorithm was used to reconstruct and quantify the vasculature. As shown in Figure 3B, the vasculature of the Control and Obesity groups was very dense, but the branches and density of the blood vessels as well as neovasculature in the PCOS-like group were significantly reduced compared to the Control group. After EA treatment, both the density of blood vessels and the density of the neovasculature were increased. As shown in Figure 4, the diameter, length, surface area, and volume of the neovasculature were all significantly decreased in both the PCOS-like and the PCOS-like-EA groups compared with the Control group. However, after the EA treatments, the length, surface area, and volume of neovascularity were significantly increased in the PCOS-like-EA group compared to the PCOS-like group.

### 2.4. EA Promotes Antral Follicle Maturation through Their Surrounding Neovasculature

**Figure 5:**
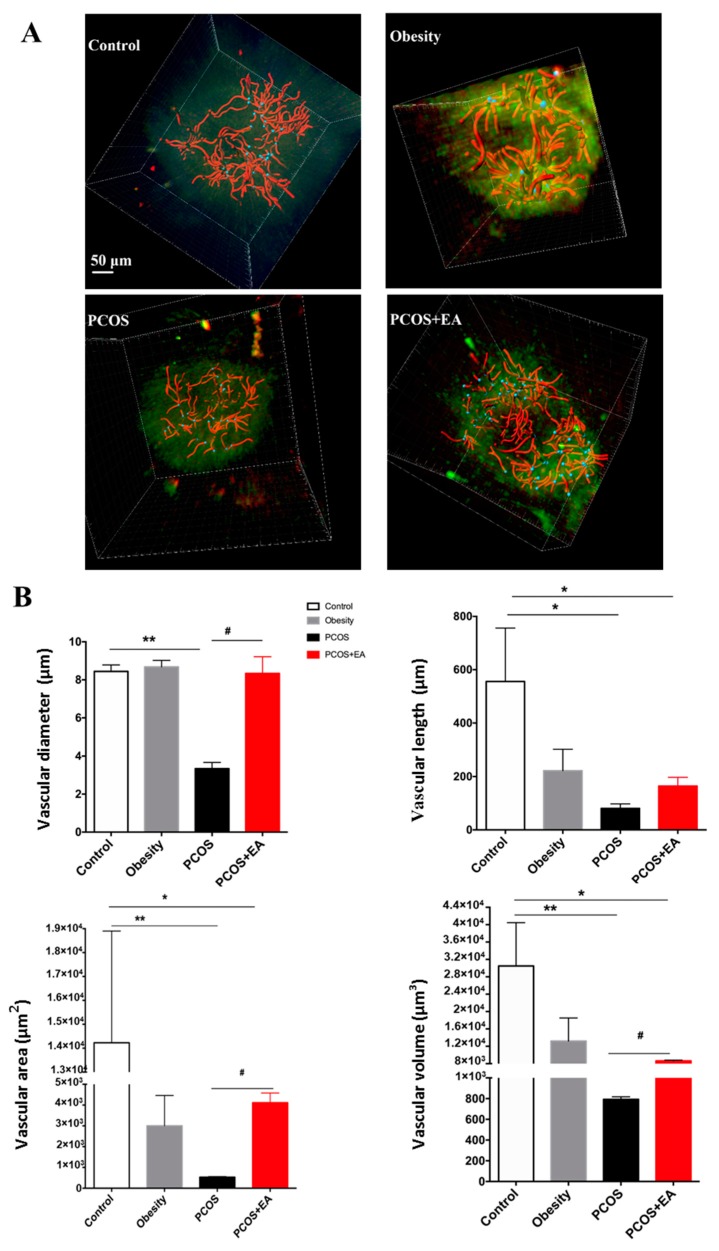
EA promotes angiogenesis surrounding the antral follicles. (**A**) Reconstruction of the neovasculature surrounding the antral follicles. (**B**) Quantification of the diameter, length, area, and volume surrounding antral follicles in the different groups. * *p* < 0.05 and ** *p* < 0.01 vs. the Control group; ^#^ *p* < 0.05 vs. the PCOS group.

Antral follicles are a critical step in the development of mature follicles. Therefore, we selected 10 antral follicles from the four groups to analyze the surrounding neovasculature. As shown in Figure 5A, the density of the neovasculature surrounding the antral follicles of the PCOS-like group was decreased compared with the other three groups. After the EA treatment, the neovasculature in the ovaries of the PCOS-like-EA group was denser than in the PCOS-like group. We further quantified the diameter, length, surface area, and volume of the neovasculature. As shown in Figure 5B, after the EA treatment, the surface area and volume of the neovasculature around the antral follicle in the PCOS-like-EA group was significantly greater than in the PCOS-like group. These data suggested that EA could directly affect the neovascularity surrounding the antral follicles under PCOS conditions.

## 3. Discussion

In the present study, we investigated the effect of ovarian vascularity on follicular maturation in a PCOS-like rat model and explored the possible mechanisms of EA. We found that DHT-induced PCOS-like rats exhibited disrupted follicular development and anovulation that were associated with alterations in the size and number of local blood vessels and the neovascularity surrounding the follicles. Low-frequency EA at the “Sanyinjiao” (ST29) and “Guilai” (SP6) acupoints directly promoted ovarian angiogenesis and neovascularity surrounding the antral follicles and partly restored ovulation.

PCOS is a complex of endocrine and metabolic disorders of uncertain aetiology in women of reproductive age [1], and it is characterized by chronic anovulation and hyperandrogenism as well as an increase in the numbers of abnormal antral follicles [2]. Acupuncture, as a natural therapy, has a long history in the treatment of gynaecological disorders in China, and in some Western countries acupuncture has been used to treat female reproductive system diseases since the end of the last century. Acupuncture has been shown to exert effects through the hypothalamic-pituitary-ovary axis [12] through central regulation of the neuroendocrine system [19] and through direct effects on the ovaries [20], adrenal glands [21], fat tissue [22], and muscles [23]. Our studies and others have shown that 3–5 treatments with low-frequency EA partially restores the oestrous cycle in DHT-induced PCOS-like rats [19] and that low-frequency EA (2 Hz, 3 mA/6 mA, 1 min) can significantly increase the ovarian blood flow velocity in PCOS-like rats, which can be maintained for 5–10 min after EA [20]. This suggests that EA can promote follicular development and ovulation in PCOS patients via both direct and indirect pathways.

Along with the animal experiments in treating PCOS with EA, we have carried out EA treatments on women with PCOS in the clinic. Considering the time and monetary cost, clinical trials have usually been designed as 30 min per treatment, twice a week for the first two weeks and then once a week for the remaining treatment, for a total of 10–14 treatments, and such treatments have an ovulation success rate of about 38% [24]. In a recent multicenter double-blind randomized controlled clinical trial, active EA treatment in 250 patients with PCOS also involved EA for 30 min per session, twice a week, with the longest treatment being 32 weeks [18]. The efficacy of treatment is closely related to the frequency of treatment, and if more intensive treatment is available the effect of EA might be faster and more consistent.

We did not use transcutaneous electro-acupuncture stimulation (TEAS) in patients with PCOS so as to compare TEAS with EA, but TEAS has been used in relieving pain [18], helping with withdrawal from smoking and drugs [25], and preventing vomiting after chemotherapy [26], and it has been shown to act mainly on the opioid systems. PCOS, as a complicated endocrine and metabolic syndrome, requires the activation and coordination of multiple targets and multiple systems. Low-frequency EA has been shown to regulate the hypothalamus-pituitary-ovary axis, to restore steroid balance, to increase insulin sensitivity, and to modulate the stimulated sympathetic nervous system. In our previous studies, we compared the effect of 2 Hz EA with 100 Hz EA [20] and the effect of different groups of acupoints [24]. The results of those studies suggest that the parameters in the present study were specific for ovulation in patients with PCOS. Therefore, the professional diagnosis and treatment of PCOS is desirable.

Some studies have investigated the role of vascular-related factors in PCOS patients [27]. Abnormal expression of multiple vascular growth factors has been seen in the ovaries of PCOS patients, and VEGF, angiopoietin-1 and 2 [28], PDGF [29], TGF-β, and bFGF are all involved in the pathophysiology of PCOS [7]. In PCOS patients, hyper-vascularized and excessive blood vessels are seen in the membrane cell layer and in the interstitium of ovarian cystic follicles [8,9]. The expression of VEGF is also significantly increased in the follicular stroma of PCOS patients [10]. When a VEGF trap (a VEGF antagonist) was administered to a normal female rhesus monkey in the middle of the follicular phase, ovulation was delayed. This led to the development of antral follicles, and the process of atresia was very similar to the pathological mechanism of PCOS [30]. In the DHEA-induced PCOS rat model, the levels of VEGF in the circulation and ovary are increased, but the expression of the VEGF receptor FLK1 is significantly lower than that of the control group. If rats are given an inhibitor of VEGF, they have reduced numbers of cystic follicles [11]. These results indicate that cystic follicles will form when VEGF family enzymes, and thus angiogenesis, are dysfunctional.

Our study used DHT (a non-aromatized androgen that excludes the effect from endogenous oestrogen) to induce PCOS symptoms, and we showed increased numbers of cystic follicles with low vascularity and reduced neovascularization surrounding the follicles, which indicated disorders in angiogenesis and developing follicles in the ovaries of PCOS-like rats. However, there have been contradictory results with hyper-vascularization in PCOS patients due to low oestrogen and progesterone when modelling, and thus the specific mechanism behind such effects needs to be further studied. The two-dimensional histologic sectioning used in traditional morphologic studies destroys the integrity of the vascular network, and thus it has been difficult to describe and analyze the relationship between ovarian vascularity and follicular development. In 2013, Karl Deisseroth of Stanford University developed a new experimental method called CLARITY [31], which uses the chemical transformation of intact biological tissues into a hydrogel-tissue hybrid that is amenable to light microscopy and the use of macromolecular labels while retaining the fine tissue structures and native biological molecules [32]. The method allows the use of immunofluorescence and in situ hybridization to label protein and nucleic acid targets after the tissue becomes transparent. Confocal microscopy and 3D analysis software can then be used to observe and analyze the target tissues and to study the complex forms and relationships between intact tissues. CLARITY thus has significant advantages for analyzing and quantifying ovarian vascularity compared to traditional two-dimensional histologic sections.

In this work, we used the CLARITY method to reconstruct the vasculature and neovasculature in whole ovaries as well as the specific follicles. The length, surface area, and volume of the vasculature and neovasculature were increased in PCOS-like rats by EA treatment along with increased numbers of mature follicles and CL (Figure 4). The key stage of follicular development is the antral follicle, and this is where major pathological changes are seen in PCOS patients. Thus we used the Crop 3D tool in the Imaris software to reconstruct the neovasculature around the antral follicles in the four groups of rats. Antral follicles can be divided into eight developmental stages. When the diameter of a follicle reaches 15–25 mm, it is considered to be in the eighth stage of follicle development and is referred to as a preovulatory follicle (mature follicle). As shown in Figure 5B, after the EA treatment the surface area and volume of the neovasculature around the antral follicles in the PCOS-like-EA group were both significantly increased compared to the PCOS-like group, which indicated that EA had a direct effect on the neovasculature surrounding the antral follicles and promoted its development and maturation. These results provide more evidence for the potential effectiveness of EA in treating PCOS.

## 4. Materials and Methods

### 4.1. Animals

Female Wistar rats (aged 3 weeks, Shanghai SLAC Laboratory Animal Co. Ltd., Shanghai, China) were housed under controlled conditions (21–22 °C, 55–65% humidity, 12 h light/12 h dark, and free access to food and water). All rats were randomly divided into four groups (Control, Obesity, PCOS-like, and PCOS-like-EA, n = 7/group). The Control, PCOS-like, and PCOS-like-EA groups were fed with normal chow (Shanghai SLAC Laboratory Animal Co. LTD, Shanghai, China), while the Obesity group was fed a high-fat chow containing 60% fat, 20% carbohydrates, and 20% protein (Open Source Diet, New Brunswick, NJ, USA). The use of animals and the experimental design were approved by the Animal Ethics Committee, School of Basic Medical Sciences of Fudan University, China (ID: 20160225-013, approval date: 25 February 2016).

### 4.2. Establishment of the PCOS-Like Rat

Based on the results of our preliminary experiments, silicone tubes with DHT (15 mg, slow releasing for 75 days, 1 cm length, 2 mm diameter) were implanted subcutaneously into the neck of the PCOS-like and PCOS-like-EA groups. Rats were anaesthetized with isoflurane, the flabby skin of the neck was separated, the tube was carefully imbedded, and the wound was sutured. Body weight, vaginal opening, and oestrus cycle were checked daily and recorded throughout the experiment.

### 4.3. Low-Frequency EA

Rats in the PCOS-like-EA group were treated with low-frequency EA from Monday to Friday for 4 weeks (for a total of 20 treatments). Under short anaesthesia, rats were inserted with single-use sterile acupuncture needles (Suzhou Shenlong Medical Apparatus Co., Ltd., Suzhou, China) into the bilateral acupoints “Guilai” (ST29) and “Sanyinjiao” (SP6). Handmade restraints were used to hold the rats in place and to maintain their posture. Acupuncture needles were inserted to a depth of 0.5–0.8 cm in the posterior part of the medial tibia for SP6 and in the bilateral part below the umbilical for ST29. The needles were attached to an electrical stimulator (HANS-LH202, Huawei Co., Ltd., Beijing, China). The treatment duration was 15 to 20 min during the first week and 30 min for the remaining treatments. The parameters for the EA treatment were 2 Hz at 2–3 mA.

### 4.4. CLARITY Approach

Rats were anesthetized i.p. with 2% sodium pentobarbital (30 mg/kg body weight, Sigma, Sigma-Aldrich; Merck Millipore, Darmstadt, Germany). We first perfused the rats with 200 mL ice-cold 1× PBS (Invitrogen GIBCO), followed by 200 mL of ice-cold hydrogel solution with 4% (weight/volume) paraformaldehyde (Rongbai Biologic Technology Co., Shanghai, China), 4% acrylamide (Sigma), 0.05% bis-acrylamide (Bio-Rad Laboratories, Hercules, CA, USA), 0.25% VA-044 (Wako Pure Chemical Industries, Ltd., Osaka, Japan), and 0.05% saponin (Sigma) in 1× PBS. After perfusion, the ovaries were extracted and immersed immediately into 10 mL hydrogel. The reaction tubes were placed at 4 °C for 3-day post-fixation, after which the oxygen was removed and the tissues were incubated for 3 h at 37 °C to polymerize the hydrogel. Hydrogel-embedded samples were then transferred into 10 mL of the clearing solution (200 mM boric acid and 4% (wt/vol) sodium dodecyl sulphate (SDS) (Sigma) dissolved in distilled H_2_O, with the addition of NaOH (EMD) to adjust to pH 8.5). The clearing solution was replaced 2–3 times/week. The rat ovaries gradually became transparent after 8–12 weeks of passive clearing on a shaking table inside a 37 °C incubator.

### 4.5. Immunofluorescence Staining

Cleared ovaries were transferred to 10 mL 1× PBST (PBS with 0.1% Triton-X100) for 1 day to remove the residual SDS. Samples were then incubated with primary antibodies (tyrosine hydroxylase 1:100 dilution, ab76442, Abcam; platelet endothelial cell adhesion molecule 1 (PECAM-1, CD31) 1:20 dilution, ab119339, Abcam; and proliferating cell nuclear antigen (PCNA) 1:50 dilution, ab18197, Abcam) for 3 days, washed in 1× PBST for 1 day, and incubated with secondary antibodies (Alexa Fluor 488 and Alexa Fluor 594 IgG, Sigma–Aldrich, Steinheim, Germany) for another 3 days in the dark. Three hours before scanning, the samples were incubated in sRIM solution (70% sorbitol in 2× PBS with 0.2% sodium azide) to correct the refractive index. All procedures were conducted at 37 °C with shaking.

### 4.6. Digital Imaging and Data Analysis

A Lightsheet Z.1 confocal microscope (Zeiss, Oberkochen, Germany) was used to collect raw images. For each imaging, one ovary was stuck on the connecting holder with glue. A 5× objective lens was set up, and the working distance was 3 mm. In the “Z-stack” tool, we could set the Z-axis of the ovary, and the “Multiview-Setup” tool was used for setting the X-axis and Y-axis. In order to get a better-quality image, the overlap parameter was set at 20%. After scanning, the data were saved as CZI format. Arivis software (Arivis AG, München, Germany) was used to compress the data and transform it into TIFF format, and Imaris software (v.8.0, Bitplane, Zurich, Switzerland) was used to analyse and reconstruct the digital images. The identity of the follicles was determined using the Imaris spot algorithm semi-manually, and the filament algorithm was used to reconstruct the shape of the vasculature [6].

### 4.7. Data Analysis

Statistical analysis was performed in GraphPad Prism (v7.0a, GraphPad Software, Inc., San Diego, CA, USA). One-way ANOVA with post-hoc Tukey’s test was used for calculating significance between groups. All data are represented as the mean ± standard error of the mean, and *p* < 0.05 was considered to be statistically significant.

## 5. Conclusions

In conclusion, the present study suggests that the follicular dysplasia seen in PCOS patients and the possible peripheral mechanism of EA might be highly related to ovarian angiogenesis. In the future, we will focus on the specific mechanisms of cytokines and signal transduction pathways induced by EA for improving angiogenesis and the potential for combining EA with other angiogenic treatments for PCOS.

## Abbreviations

PCOS	Polycystic ovary syndrome
EA	Electro-acupuncture
DHT	5α-dihydrotestosterone
CL	Corpus luteum
VEGF	Vascular endothelial growth factor
PDGF	Platelet derived growth factor
TGF-β	Transforming growth factor beta
bFGF	Basic fibroblast growth factor
PECAM-1/CD31	Platelet endothelial cell adhesion molecule 1
PCNA	Proliferating cell nuclear antigen
DHEA	dehydroepiandrosterone

## References

[1] Li R., Zhang Q., Yang D., Li S., Lu S., Wu X., Wei Z., Song X., Wang X., Fu S. (2013). Prevalence of polycystic ovary syndrome in women in China: A large community-based study. Hum. Reprod..

[2] March W.A., Moore V.M., Willson K.J., Phillips D.I., Norman R.J., Davies M.J. (2010). The prevalence of polycystic ovary syndrome in a community sample assessed under contrasting diagnostic criteria. Hum. Reprod..

[3] Michelmore K.F., Balen A.H., Dunger D.B., Vessey M.P. (2010). Polycystic ovaries and associated clinical and biochemical features in young women. Clin. Endocrinol..

[4] Yildiz B.O., Bozdag G., Yapici Z., Esinler I., Yarali H. (2012). Prevalence, phenotype and cardiometabolic risk of polycystic ovary syndrome under different diagnostic criteria. Hum. Reprod..

[5] Ferraretti A.P., Marca A.L., Fauser B.C.J.M., Tarlatzis B., Nargund G., Gianaroli L. (2011). ESHRE consensus on the definition of ‘poor response’ to ovarian stimulation for in vitro fertilization: The Bologna criteria. Hum. Reprod..

[6] Feng Y., Cui P., Lu X., Hsueh B., Möller B.F., Zarnescu Y.L., Tomer R., Boerboom D., Carmeliet P., Deisseroth K. (2017). CLARITY reveals dynamics of ovarian follicular architecture and vasculature in three-dimensions. Sci. Rep..

[7] Tal R., Seifer D.B., Arici A. (2015). The emerging role of angiogenic factor dysregulation in the pathogenesis of polycystic ovarian syndrome. Semin. Reprod. Med..

[8] Abbott D.H., Dumesic D.A., Franks S. (2002). Developmental origin of polycystic ovary syndrome—A hypothesis. J. Endocrinol..

[9] Pan H.A., Wu M.H., Cheng Y.C., Li C.H., Chang F.M. (2002). Quantification of Doppler signal in polycystic ovary syndrome using three-dimensional power Doppler ultrasonography: A possible new marker for diagnosis. Hum. Reprod..

[10] Brown L.F., Tognazzi K., Dvorak H.F., Harrist T.J. (1996). Strong expression of kinase insert domain-containing receptor, a vascular permeability factor/vascular endothelial growth factor receptor in AIDS-associated Kaposi’s sarcoma and cutaneous angiosarcoma. Am. J. Pathol..

[11] Abramovich D., Irusta G., Bas D., Cataldi N.I., Parborell F., Tesone M. (2012). Angiopoietins/TIE2 system and VEGF are involved in ovarian function in a DHEA rat model of polycystic ovary syndrome. Endocrinology.

[12] Feng Y., Johansson J., Shao R., Holm L.M., Billig H., Stener-Victorin E. (2012). Electrical and manual acupuncture stimulation affect oestrous cyclicity and neuroendocrine function in an 5α-dihydrotestosterone-induced rat polycystic ovary syndrome model. Exp. Physiol..

[13] Kokosar M., Benrick A., Perfilyev A., Nilsson E., Källman T., Ohlsson C., Ling C., Stenervictorin E. (2018). A Single Bout of Electroacupuncture Remodels Epigenetic and Transcriptional Changes in Adipose Tissue in Polycystic Ovary Syndrome. Sci. Rep..

[14] Wang S., Zhang J., Yang H., Wang F., Li S. (2015). Acupoint specificity on acupuncture regulation of hypothalamic- pituitary-adrenal cortex axis function. BMC Complement. Altern. Med..

[15] Kuang H., Li Y., Wu X., Hou L., Wu T., Liu J., Ng E.H.Y., Stenervictorin E., Legro R.S., Zhang H. (2013). Acupuncture and Clomiphene Citrate for Live Birth in Polycystic Ovary Syndrome: Study Design of a Randomized Controlled Trial. Evid.-Based Complement. Altern. Med..

[16] Smith C.A., Armour M., Ee C. (2016). Complementary Therapies and Medicines and Reproductive Medicine. Semin. Reprod. Med..

[17] Stener-Victorin E., Jedel E., Mannerås L. (2010). Acupuncture in Polycystic Ovary Syndrome: Current Experimental and Clinical Evidence. J. Neuroendocr..

[18] Wu X.K., Stenervictorin E., Kuang H.Y., Ma H.L., Gao J.S., Xie L.Z., Hou L.H., Hu Z.X., Shao X.G., Ge J. (2017). Effect of Acupuncture and Clomiphene in Chinese Women With Polycystic Ovary Syndrome: A Randomized Clinical Trial. JAMA.

[19] Feng Y., Johansson J., Shao R., Mannerås L., Fernandez-Rodriguez J., Billig H., Stener-Victorin E. (2009). Hypothalamic neuroendocrine functions in rats with dihydrotestosterone-induced polycystic ovary syndrome: Effects of low-frequency electro-acupuncture. PLoS ONE.

[20] Stener-Victorin E., Kobayashi R., Kurosawa M. (2003). Ovarian blood flow responses to electro-acupuncture stimulation at different frequencies and intensities in anaesthetized rats. Auton. Neurosci. Basic Clin..

[21] Zhao Z.L., Kim S.C., Zhang J., Liu H.F., Lee B.H., Jang E.Y., Lee C.W., Cho I.J., An W.G., Yang C.H. (2016). Hypothalamic Norepinephrine Mediates Acupunctural Effects on Hypothalamic-Pituitary-Adrenal Axis During Ethanol Withdrawal. J. Acupunct. Meridian Stud..

[22] Johansson J., Manneråsholm L., Shao R., Olsson A., Lönn M., Billig H., Stenervictorin E. (2013). Electrical vs Manual Acupuncture Stimulation in a Rat Model of Polycystic Ovary Syndrome: Different Effects on Muscle and Fat Tissue Insulin Signaling. PLoS ONE.

[23] Johansson J., Feng Y.R., Lonn M., Billig H., Stener V.E. (2010). Intense electroacupuncture normalizes insulin sensitivity, increases muscle GLUT4 content, and improves lipid profile in a rat model of polycystic ovary syndrome. Am. J. Physiol. Endocrinol. Metab..

[24] Stener-Victorin E., Waldenstrom U., Tagnfors U., Lundeberg T., Lindstedt G., Janson P.O. (2000). Effects of electro-acupuncture on anovulation in women with polycystic ovary syndrome. Acta Obstet. Gynecol. Scand..

[25] Lambert C., Berlin I., Lee T.L., Hee S.W., Tan A.S., Picard D., Han J.S. (2011). A standardized transcutaneous electric acupoint stimulation for relieving tobacco urges in dependent smokers. Evid.-Based Complement. Altern. Med..

[26] Zhang X., Jin H.F., Fan Y.H., Lu B., Meng L.N., Chen J.D. (2014). Effects and mechanisms of transcutaneous electroacupuncture on chemotherapy-induced nausea and vomiting. Evid.-Based Complement. Altern. Med..

[27] Assila B.S., Fatma M., Olfa K., Malek S., Faten H.B.A., Sondes H., Faouzi J., Mounir A., Muhammad A.E., Mourad A. (2016). Vascular endothelial growth factor (VEGFA) gene variation in polycystic ovary syndrome in a Tunisian women population. BMC Genom..

[28] Di Pietro M., Parborell F., Irusta G., Pascuali N., Bas D., Bianchi M.S., Tesone M., Abramovich D. (2015). Metformin regulates ovarian angiogenesis and follicular development in a female polycystic ovary syndrome rat model. Endocrinology.

[29] Pietro M.D., Scotti L., Irusta G., Tesone M., Parborell F., Abramovich D. (2016). Local administration of platelet-derived growth factor B (PDGFB) improves follicular development and ovarian angiogenesis in a rat model of Polycystic Ovary Syndrome. Mol. Cell. Endocrinol..

[30] Fraser H.M., Wilson H., Rudge J.S., Wiegand S.J. (2005). Single injections of vascular endothelial growth factor trap block ovulation in the macaque and produce a prolonged, dose-related suppression of ovarian function. J. Clin. Endocrinol. Metab..

[31] Chung K., Wallace J., Kim S.Y., Kalyanasundaram S., Andalman A.S., Davidson T.J., Mirzabekov J.J., Zalocusky K.A., Mattis J., Denisin A.K. (2013). Structural and molecular interrogation of intact biological systems. Nature.

[32] Tomer R., Ye L., Hsueh B., Deisseroth K. (2014). Advanced CLARITY for rapid and high-resolution imaging of intact tissues. Nat. Protoc..

